# External and internal shell formation in the ramshorn snail *Marisa cornuarietis* are extremes in a continuum of gradual variation in development

**DOI:** 10.1186/1471-213X-13-22

**Published:** 2013-05-17

**Authors:** Leonie Marschner, Julian Staniek, Silke Schuster, Rita Triebskorn, Heinz-R Köhler

**Affiliations:** 1 Animal Physiological Ecology, Institute of Evolution and Ecology, University of Tübingen, D-72072 Tübingen, Germany; 2 Transfer Center for Ecotoxicology and Ecophysiology, D-72108 Rottenburg, Germany

**Keywords:** Platinum, Shell Internalization, Embryogenesis, Mantle Formation

## Abstract

**Background:**

Toxic substances like heavy metals can inhibit and disrupt the normal embryonic development of organisms. Exposure to platinum during embryogenesis has been shown to lead to a “one fell swoop” internalization of the shell in the ramshorn snail *Marisa cornuarietis*, an event which has been discussed to be possibly indicative of processes in evolution which may result in dramatic changes in body plans.

**Results:**

Whereas at usual cultivation temperature, 26°C, platinum inhibits the growth of both shell gland and mantle edge during embryogenesis leading to an internalization of the mantle and, thus, also of the shell, higher temperatures induce a re-start of the differential growth of the mantle edge and the shell gland after a period of inactivity. Here, developing embryos exhibit a broad spectrum of shell forms: in some individuals only the ventral part of the visceral sac is covered while others develop almost “normal” shells. Histological studies and scanning electron microscopy images revealed platinum to inhibit the differential growth of the shell gland and the mantle edge, and elevated temperature (28 - 30°C) to mitigate this platinum effect with varying efficiency.

**Conclusion:**

We could show that the formation of internal, external, and intermediate shells is realized within the continuum of a developmental gradient defined by the degree of differential growth of the embryonic mantle edge and shell gland. The artificially induced internal and intermediate shells are first external and then partly internalized, similar to internal shells found in other molluscan groups.

## Background

The phenotype of an organism is not only determined by its genotype but also by the regulation of gene activity. Gene regulation can be influenced by environmental factors like temperature or presence of chemicals and thus lead to the emergence of new phenotypic traits [1]. Platinum, acting on the embryonic development of the ramshorn snail *Marisa cornuarietis* has been shown to induce severe changes in the body plan [2,3]. As described by Marschner *et al.*[3], mantle and shell gland, tissues that normally spread across the visceral sac and cover it, stop growing during embryogenesis and remain on the ventral side of the visceral sac which itself has rotated horizontally by 90°. There, calcium carbonate is formed inside of the snail’s body, thus forming an internal shell instead of an external one. Gastropods, in contrast to other molluscs, are characterized by an event called “torsion”, a horizontal rotation of the visceropallium relative to head and foot by 180°, leading to a change of their internal anatomy [4-6]. In *Marisa cornuarietis*, this torsion can actually be observed during embryonic development as a rotation of the visceral sac due to the differential growth of the mantle tissue on its left side [7]. However, under the influence of platinum, this differential growth is stopped and a rotation does not occur. Nevertheless, these platinum-exposed individuals display several traits that are usually attributed to the process of torsion. They show an anterior anus, the ctenidium, however, is positioned on the posterior part of the visceral sac and not above the head. Both the ctenidium and the osphradium are even further displaced by the vertical rotation of the visceropallium. Those organs rotate sinistrad and the ctenidium in these snails becomes positioned on the left dorso-lateral side of the visceral sac. The osphradium, which normally can be found in the mantle cavity, is even shifted to the left ventro-lateral side of the visceral sac. Platinum-exposed embryos do not have an external mantle and a mantle cavity does not develop. Despite its new position, the shell-secreting tissue on the ventral side of the visceral sac secretes calcium carbonate which forms an internal shell cupping the digestive gland. These “sluggish” snails show traits of both “torted” (anterior anus) and “untorted” (no horizontal rotation of the visceropallium, posterior ctenidium) molluscs [8,9]. Assuming that the rotation in *Marisa* is similar to the ontogenetic torsion that can be observed in other gastropods, it seems that gastropod characteristics that are supposedly caused by a torsion-like rotation of the visceropallium, at least in *Marisa*, might be independent from a rotation and, consequently, from each other.

However, the mechanism behind this platinum-induced developmental aberration is not yet known, but since platinum obviously affects development-controlling processes, we investigated whether platinum interacts with elevated temperature which is known to influence development as well, particularly in ectotherms. We exposed *Marisa cornuarietis* embryos to both platinum and elevated temperatures 2 to 4°C higher than in previous experiments with this species [2,3,10-13]. In the first preliminary studies with combinations of platinum and elevated temperature we observed a new kind of developmental aberration which, at first sight, seems to be an “intermediate” between the already described “sluggish” snails which develop internal shells after platinum exposure and “normal” control snails. We called these animals “partly-shelled”, since they developed a partial external shell, covering the ventral part of the visceral sac but which often do not extend to the dorsal part.

In order to elucidate the differences in the development of the “partly-shelled” animals and those with internal shells, we investigated adult “sluggish” and control individuals as well as “partly-shelled” and normally developing embryos histologically. As well, the course of development of platinum-plus-heat-exposed embryos was studied by scanning electron microscopy.

## Results

**Figure 1:**
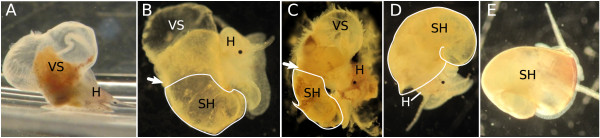
***Marisa cornuarietis *individuals after hatching with different types of shells.** Arrows indicate the mantle edge, “partial” shells are encircled in white; **A**: “sluggish” snail without an external mantle and with an internal shell (about 3 weeks old); **B**, **C**: snails with “partial” shells, **D**: snail with an almost normally developed “partial” shell; **E**: normally developed snail from the control; H: head, SH: shell, VS: visceral sac.

Figure 1 shows selected individuals which have developed the different types of shells that we found in our experiments, ranging from completely “shell-less” or “sluggish”, i.e. snails with fully internal shells (Figure 1A), and “partly-shelled” individuals with small external shells, covering varying parts of the visceral sac (Figure 1B-D), to normally shelled animals (Figure 1E).

The normal embryonic development of Marisa cornuarietis has been thoroughly described by Demian and Yousif [7,14-17]. An analysis of the embryonic development of platinum-exposed “sluggish” snails can be found in Marschner et al. [3]. All this work will be used to compare the development of “partly-shelled” snails with.

### Differential growth of mantle anlage, mantle edge, and shell gland in “sluggish” snails

**Figure 2:**
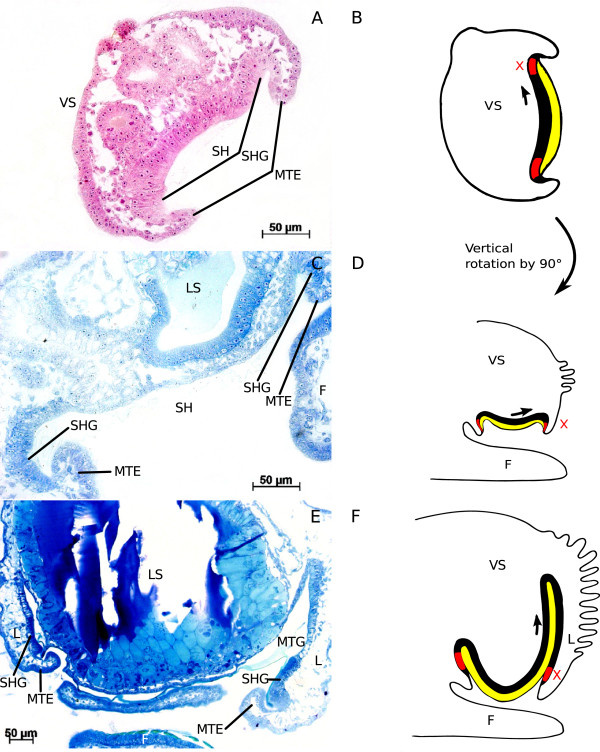
**Histological sections of Pt-exposed embryos and respective sketches explaining derection of tissue growth.** Arrows indicate directional growth of a tissue, X indicates stop of growth of the respective tissue; black: mantle tissue, red: shell gland, yellow: shell; **A**: 3-day-old embryo, transverse section through the visceral sac; **B**: sketch of Figure 2A, frontal view; **C**: 7-day-old embryo, sagittal section; **D**: sketch of Figure 2C, left lateral view; **E**: 9-day-old embryo, transverse section through the visceral sac; **F**: sketch of Figure 2E, left lateral view; F: foot; L: lobe; LS: larval stomach; MTE: mantle edge; MTG: mantle gap; SH: shell; SHG: shell gland; VS: visceral sac.

Figure 2 displays sections of Pt-exposed embryos from the study of Marschner et al. [3] and sketches of these sections to illustrate development. Pt-exposed embryos develop similarly to control embryos until the onset of torsion. However, when torsion is supposed to begin by differential growth of the mantle anlage (i.e. tissue proximate to the protoconch in “sluggish” individuals), mantle edge, and shell gland, no differential growth of these tissues is visible after Pt-exposure. In contrast, these structures at first remain on the left side of the visceral sac and do not grow much (Figure 2A, B) but then shift to the ventral side of the visceral sac due to a vertical rotation of the visceral sac by 90° (Figure 2C, D) [3]. In this position, the shell gland continues to secrete calcium carbonate. A closer examination of the developmental states shown in Figure 2 makes evident that, at the beginning, the shell gland is located on the visceral sac itself (Figure 2A, B). As can be taken from Demian and Yousif’s [16] description of the “normal” development of shell gland and mantle, the shell gland remains on the surface of the visceral sac, encircling the mantle. The mantle edge is a fold that partially covers the shell gland. In Pt-exposed, “sluggish” embryos, however, the shell gland moves from the visceral sac’s surface to the proximal side of a lobe protruding from the visceral sac (Figure 2C, D). In this process a “mantle gap” between lobe and the shell-secreting tissue covering the digestive gland develops.

**Figure 3:**
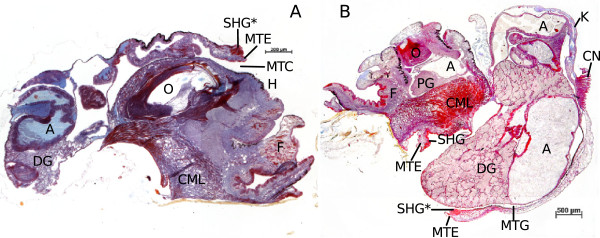
**Sagittal sections of adult *M. cornuarietis*.** Asterisks indicate corresponding structures; **A**: snail from the control, six weeks after oviposition, right lateral view; **B**: Pt-exposed snail, several months old, left lateral view; A: alimentary tract; CML: columellar muscle; CN: ctenidium; DG: digestive gland; F: foot; K: kidney; MTC: mantle cavity; MTE: mantle edge; MTG: mantle gap; O: odontophor; PG: pedal ganglion; SHG: shell gland.

During further growth this lobe increases in length, and both mantle edge and shell gland move further away from the visceral sac (Figure 2E, F). This affects also the position of mantle edge and shell gland which become located on the inner side of the lobe (Figure 2). These observations suggest that the mantle anlage does not actually stop growing. It is solely the complex of shell gland and mantle edge that does not increase in diameter. The tissue that is encircled by mantle edge and shell gland is in fact the tissue that would normally cover the visceral sac forming the mantle in the conventional way. The direction of growth of this mantle tissue, however, is restricted in the “sluggish” individuals and directed by the unyielding tissues of mantle edge and shell gland which form a kind of inflexible ring around the growing mantle tissue, forcing it to fold inwards (Figure 2E, F). These diverging forms of development are also illustrated in Figure 3, which shows a control snail (Figure 3A) with the shell gland in its usual position on the visceral sac, facing away from the snail’s body. In contrast, Figure 3B shows an adult “sluggish” snail whose shell gland faces the interior of the mantle gap. In the case of Pt-exposed snails, it forms a part of the inner lining of the lobe where it encircles the mantle tissue which also forms the lining of the lobe and covers the visceral sac’s surface distal of the digestive gland. Those snails do not only possess an internal shell as was first noticed by Osterauer et al. [2], they also have an internal mantle.

### Embryonic development of “partly-shelled” snails

**Figure 4:**
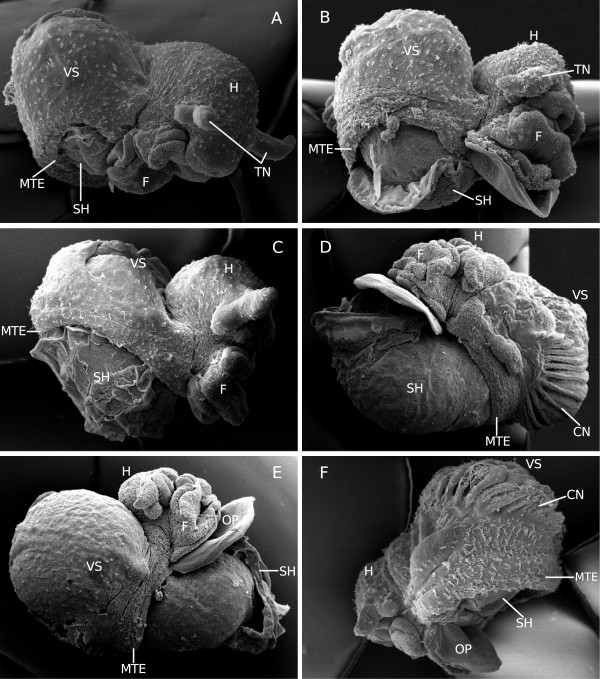
**SEM-images of *M. cornuarietis *embryos from the same clutch (A-E) exposed to platinum and elevated temperature at different stages of development. A**: seven-day-old embryo resembling a stage of development present also in “sluggish” individuals as described in Marschner et al. [3], right lateral view; **B**: eight-day-old embryo, right lateral view; **C**: nine-day-old embryo, right lateral view; **D**: ten-day-old embryo, left lateral view; **E**: eleven-day-old embryo, right lateral view; **F**: nine-day-old embryo from a different clutch, left lateral view; CN: ctenidium; F: foot; H: head; MTE: mantle edge; OP: operculum; SH: shell; TN: tentacles; VS: visceral sac.

The embryonic development of M. cornuarietis in the presence of platinum and at elevated temperature is shown in Figure 4. All embryos, except for the one shown in Figure 4F, derive from the same clutch. Since they were fixed on subsequent days, Figure 4A-E show a developmental sequence. The sequence starts with an embryo at the age of 7 days (Figure 4A). This embryo resembles the appearance of the “sluggish” embryos that have been described by Marschner et al. [3] at approximately the same developmental stage as “sluggish” embryos of similar age, although it is a bit further developed due to the higher cultivation temperature. Subsequently, however, their development begins to differ from the development of platinum-exposed embryos at 26°C. At lower temperatures, morphogenesis is completed as soon as shell gland and mantle edge have stopped growing and the visceral sac has rotated horizontally. In contrast to this, the snails that are exposed to platinum at higher temperatures experience a new growth impulse that leads to a gradual covering of the visceral sac by a cap-like external shell while mantle edge and shell gland slowly grow craniad (Figure 4B-E). The onset of this new growth impulse can vary slightly between different clutches as shown in Figure 4F which displays a nine-day-old embryo that does not yet exhibit any signs of a formation of a “partial-shell” unlike its contemporary in Figure 4C.

**Figure 5:**
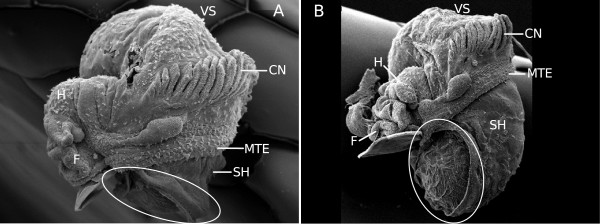
**Images of platinum-plus-heat-exposed embryos.** Left lateral views, circles highlight the portion of calcium carbonate that was secreted before the re-start of the differential growth of the shell gland-mantle edge-complex; **A**: 7-day-old embryo; **B**: 10-day-old embryo; CN: ctenidium; F: foot; H: head; MTE: mantle edge; SH: shell; VS: visceral sac.

Although shell gland and mantle edge do not grow much in “sluggish” embryos, calcium carbonate nevertheless is secreted [3]. While the shell gland is positioned on the ventral side of the visceral sac, it secretes calcium carbonate which accumulates on the mantle anlage roughly in the shape of a plate which is subsequently incorporated into the developing internal shell as the mantle tissue folds inwards. However, in Pt-exposed snails at higher temperatures, mantle edge and shell gland resume growth and, thus, an external shell is formed. On the distal part of the shell, the “plate” that was formed before the re-start of the differential growth, is still visible (Figure 5A, B). In the case of the “partly-shelled” snails, the “plate” becomes part of the external shell instead of the internal shell.

### Histology of “partly-shelled” snails

**Figure 6:**
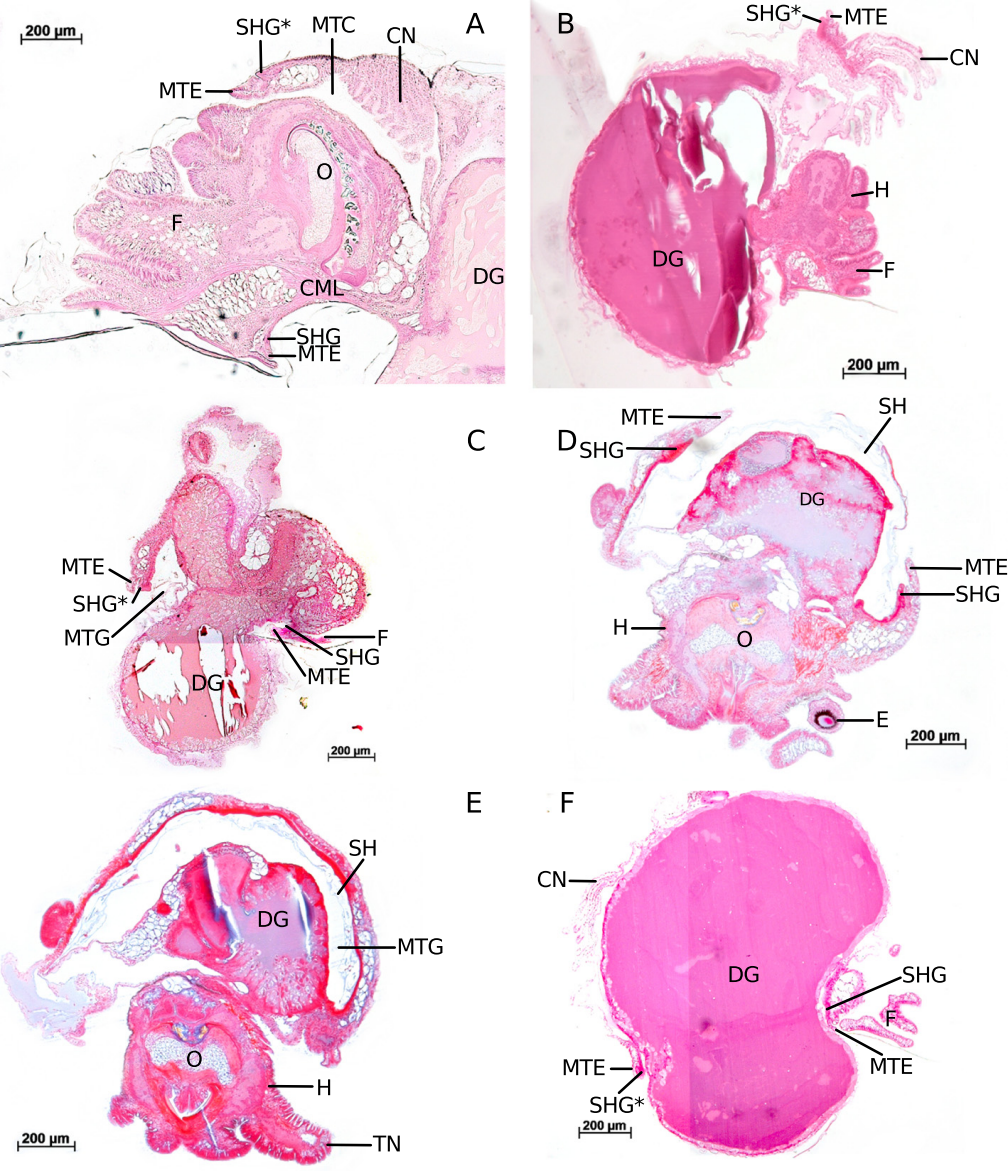
**Histological sections of *M. cornuarietis *embryos.** Asterisks indicate corresponding structures; A: Sagittal section of a cephalopodium of a control snail, 12 days after oviposition, left lateral view; B: Sagittal section of a platinum-plus-heat-exposed snail, 12 days after oviposition, right lateral view; C: Sagittal section of a platinum-plus-heat-exposed snail, 17 days after oviposition, right lateral view; D: Horizontal section of a platinum-plus-heat-exposed snail, 17 days after oviposition; E: same individuum as in D, section more dorsal; F: Sagittal section of a platinum-plus-heat-exposed snail, 12 days after oviposition; CML: columellar muscle; CN: ctenidium; DG: digestive gland; E: eye; F: foot; H: head; MTC: mantle cavity; MTE: mantle edge; MTG: mantle gap; O: odontophor; SHG: shell gland.

In Figure 6 histological sections of a control snail (Figure 6A) and several “partly-shelled” animals are shown (Figure 6B-F). Figure 6B displays a 12-day-old “partly-shelled” individual. The visceral sac in this animal is almost completely covered by an external shell, and only the ctenidium remains uncovered. The ctenidium actually has moved craniad and is now positioned above the head, a position that cannot be found in “sluggish” snails but which is normal for prosobranch gastropods as displayed by the control individual in Figure 6A. The mantle gap is basically nonexistent, and there is only a very small lobe holding shell gland and mantle edge. Figure 6C shows an older individual (17 days after oviposition) which shows a smaller external shell and a larger mantle gap than the younger individual in Figure 6B. Figures 6D and E both display horizontal sections of the same snail. In Figure 6D a section through the head and the visceral sac is shown, the section plane was slightly tilted, and mantle edge, shell gland and uncovered part of the external shell are visible. The section in Figure 6E is located dorsal to the section in Figure 6D, showing the posterior part of the digestive gland to be completely surrounded by the mantle gap and the lobe. The mantle gap also contains calcium carbonate, forming the part of the external shell that remains covered by the lobe. The “partly-shelled” snails do not only experience a new growth impuls, they also show some shell coiling (Figure 4D, E, Figure 6B, Figure 6B, C, F). However, the coils of these shells are not as tight as in control snails.

### Comparison of embryogenesis in normal, “sluggish” and “partly-shelled” M. cornuarietis

**Figure 7:**
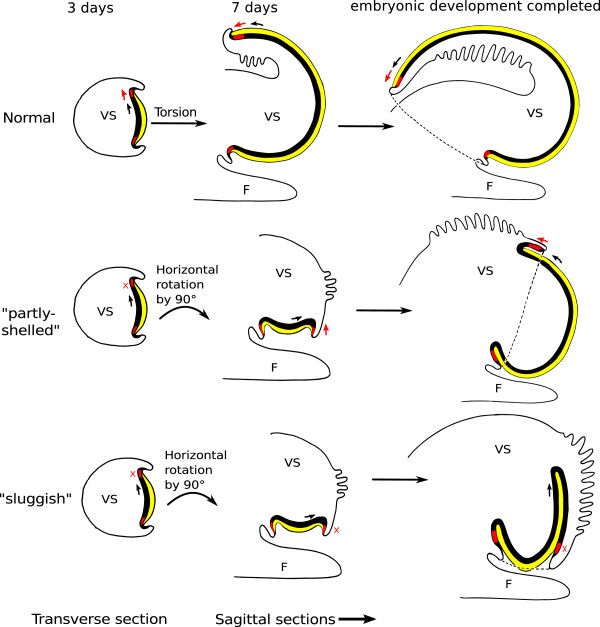
**Sketches of three different developmental stages (aged 3 (frontal view) and 7 days (left lateral view) and after completion of embryonic development (left lateral view)) of normal (top row), “partly-shelled” (middle row) and “sluggish” individuals (bottom row).** Black: mantle tissue; red: shell gland; yellow: shell, direction of growth is indicated by an arrow, arrest of growth is indicated by X; F: foot; VS: visceral sac.

**Figure 8:**
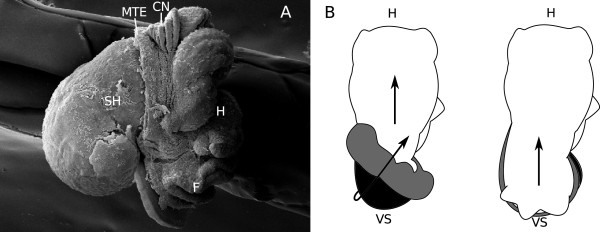
**Differential growth in “partly-shelled” snails. A**: SEM-image of a 9-day-old platinum-plus-heat-exposed animal with a well developed shell whorl (right lateral view); **B**: sketches of a control animal (left) and a “partly-shelled” animal (right), dorsal view, black: mantle tissue; grey: mantle edge; arrows indicate the direction of differential growth; CN: ctenidium; F: foot; H: head; MTE: mantle edge; SH: shell; VS: visceral sac.

In Figure 7 sketches of selected developmental stages of the differently treated snails are depicted. The top row shows the normal development [3]. At the age of three days, the mantle anlage and the shell gland are positioned on the left side of the visceral sac. During further development, differential growth of the distal parts of the mantle anlage and the shell gland leads to an overgrowing of the visceral sac and an enfolding of the epidermal tissue of the right side of the visceral sac into the mantle cavity. Thus, the shell gland moves its orientation from being located parallel to the longitudinal body axis to being located perpendicular to the axis. After this rotation, mantle, shell gland, and mantle edge keep growing; the dorsal parts of the respective tissues grow to a greater extent than the ventral parts and thus the animal coils. In “sluggish” snails, shown in the bottom row of the sketches in Figure 7, the differential growth is limited to the mantle anlage. Mantle edge and shell gland arrest growth and, therefore, do not overgrow the visceral sac. Simultaneously, however, the mantle anlage keeps growing and invaginates into the body. The arrested mantle edge and shell gland form a kind of “inelastic ring” around the mantle anlage and thus facilitate the formation of an internal shell. The mantle moulds itself around the digestive gland and even folds in again on itself, forming a lobe and encircling a mantle gap. In the “partly-shelled” animals, displayed in the middle row of sketches, however, shell gland and mantle anlage resume their growth after several days of arrest. Their shells show a slight coiling but no real spiralling. Due to the temporary arrest of shell gland and mantle edge growth, the mantle anlage has started to invaginate into the body, forming a lobe like in the “sluggish” animals. As soon as the growth of the mantle edge and the shell gland resumes, the mantle tissue grows across the visceral sac, and only a small lobe and a small mantle gap remain which both are pushed craniad by the growing mantle. This revived growth is also differential, as the distal parts of shell gland and mantle edge grow more than the proximal parts. This differential growth results in the coiling of shell and visceral sac. Usually, the shell of the “partly-shelled” snails is only slightly coiled but, occasionally, almost normal shell coiling can be observed (Figure 8). The direction of the “revived” growth of mantle tissue, shell gland and mantle edge is parallel to the longitudinal body axis as it is in normally developing M. cornuarietis whose shells are planispiral. Figure 8B illustrates the respective directions of the differential growth of mantle tissue, shell gland and mantle edge in the differently treated snails. In the control, the first direction of differential growth is angular and results in a rotation of the visceral sac (ontogenetic torsion). After the completion of torsion, the differential growth vector shifts and now points parallel to the longitudinal body axis leading to the planispiral growth of the shell. In “sluggish” snails the outer tissues, mantle edge and shell gland, stop growing altogether, and the growth of the mantle anlage is restricted to the interior of the snail. Therefore, any directional or differential growth of the mantle tissue cannot be observed from the outside and is probably obscured due to lack of space in the snail’s body. In “partly-shelled” animals, however, shell gland and mantle edge resume their growth and, since the exterior shell of these animals is also planispiral and slightly coiled, the growth vector in these animals is in line with the longitudinal body axis and not angular, starting from the ventral side of the visceral sac after its vertical rotation by 90°.

## Discussion

There have been reports on artificially induced shell internalization before. Removal of distinct micromeres during early cleavage in Ilyanassa obsoleta can lead to veliger larvae that do not develop an exterior shell but in which an internal precipitate of calcium carbonate can be found [18,19]. McCain [19] suggested that the reason for the development of an internal shell in Ilyanassa embryos is a disruption of normal inductive interactions between the calcium carbonate precursor cells (cells that regulate shell and statocyst formation) in addition to the interactions between the 3D macromere and the micromeres. Dictus and Damen [20] showed that the progeny of several micromeres contribute to the shell-secreting tissue of the larva in Patella vulgata. For Marisa cornuarietis, however, it is not known which micromeres contribute to which tissue [7] and, therefore, any speculation about a possible connection between a tissue’s sensitivity to platinum and its progenitor cells is difficult. Besides, the induction of shell internalization by deletion of micromeres happens very early in embryonic development, whereas the platinum-induced shell internalization becomes feasible only in later stages of development, caused by a different mechanism that affects tissue growth and not tissue formation.

In normally developing Marisa embryos two different directions of differential growth can be observed: first, the mantle anlage, the shell gland and the mantle edge overgrow the visceral sac in a way that leads to a horizontal rotation of the visceral sac by 180° [7]. Subsequently, differential growth of the dorsal part of mantle, shell gland, and mantle edge, results in shell coiling [21]. Torsion and coiling are independent from each other [21,22]. Marschner et al. [3] showed that ontogenetic torsion does not take place in “sluggish” Marisa snails, however, it is not clear whether it is also absent in the “partly-shelled” individuals described here. A horizontal rotation cannot be observed in “partly-shelled” snails, although, in some specimens the ctenidium was positioned above the head which is a position which is supposed to be caused by ontogenetic torsion. In the “partly-shelled” snails the mantle anlage, the shell gland, and the mantle edge rotate vertically by 90° just like in the “sluggish” conspecifics. This rotation is unrelated to torsion and its cause is unknown. However, in contrast to the “sluggish” snails, the mantle tissue, the shell gland and the mantle edge resume growth in the “partly-shelled” animals. These tissues overgrow the visceral sac from a position on the ventral side of the visceral sac and thus the growth vector is not angular to the longitudinal body axis and no rotation of the visceral sac occurs. During this process, the tissue on the dorsal side of the visceral sac is pushed craniad, including the ctenidium which is drawn closer to its “normal” position above the snails’ head. Due to the vertical rotation by 90°, the ctenidium starts its movement from the left side of the snail’s body, and since the growth vector is parallel to the longitudinal body axis, the ctenidium ends up above the snail’s head but is located still more to the left side instead of right as it is in controls. This way, a criterion that is usually attributed to torsion, the anterior ctenidium, can be formed without any horizontal rotation of the visceral sac. Comparing normally developing and exposed embryos, it can be concluded that platinum inhibits the differential growth of both shell gland and mantle edge. In the case of the Pt-exposed “sluggish” animals these tissues remain inactive and neither angular nor straight differential growth takes place. In contrast, the combination of platinum and heat first leads to the described Pt-induced arrest of tissue growth but, some time later, heat reverses the effect. However, an angular growth does not take place, and straight differential growth leads to coiling. The platinum effect on the embryonic development of Marisa cornuarietis is obviously tissue-specific in the way that platinum specifically inhibits the growth of these tissues.

Growth of tissues and organs often occurs through cell proliferation which is usually, but not always, induced by growth factors [23]. Grande and Patel [24] investigated a signalling molecule of the growth factor β superfamily named Nodal. Nodal is involved in the development of chirality in snails and its inhibition by the chemical SB-431542 can lead to a loss of chirality that results in uncoiled shells. Disruption of the Nodal pathway obviously disrupts the differential growth that leads to coiling. The morphology of the resulting phenotype differs from the one induced by platinum, but is another example for body plan modification resulting from the inhibition of differential tissue growth by a single agent. Gene expression in the shell-secreting tissue has been investigated in Haliotis asinina [25] and Patella vulgata [26]. Although many of these upregulated genes are probably involved in shell formation, the gene dpp-BMP2/4, that was traced by Nederbragt et al. [26] in the ectodermal tissue surrounding the mantle edge, is known for its role in the specification of the dorsoventral axis in vertebrates and insects [27]. Nederbragt et al. [26] hypothesized that engrailed, which is expressed in the shell-secreting tissue, and dpp-BMP2/4 together set up a compartment boundary between shell-secreting and non-secreting tissue. This hypothesis was supported by Baratte et al. [28] who found engrailed proteins at the border of the mantle edge in the shell sac of Sepia officinalis, a cephalopod with an internal shell. These findings are especially interesting since dpp-BMP2/4, like Nodal, also belongs to the transforming growth factor β superfamily [29]. Both engrailed and dpp-BMP2/4 expression should therefore be investigated in control and platinum-exposed Marisa cornuarietis in the future, in order to find out, whether the inhibition of the differential growth in platinum-exposed snails is also due to a disruption of a growth factor as it is in the case of inhibited shell coiling.

There are also several accounts on how heavy metals can influence tissue growth and development. Hanna et al. [30] investigated several heavy metals and their impact on cell proliferation in embryos. They found that heavy metals could significantly reduce the number of cells in developing mouse embryos. Heavy metals can also act as carcinogens on different stages of carcinogenesis, including mutagenesis and altering gene expression [31].

Since some organoplatinum compounds are potent anticancer drugs, the genotoxicity of these chemicals has received reasonable attention. The genotoxicity of the different organoplatinum agents varies with their valency, structure, and conformation [32]. While Gebel *et al*. [32] did not observe any induction of micronuclei in human peripheral lymphocytes by PtCl_2_, Migliore et al. [33] found a significant increase of micronuclei in human lmyphocytes. According to Nordlind [34], PtCl_2_ inhibits the DNA synthesis of human lymphoid cells. Also, Osterauer et al. [35] observed a significant increase in DNA damage induced by PtCl_2_ in *Marisa cornuarietis*. It is, however, unlikely that genotoxicity alone can explain the highly tissue specific action of PtCl_2_ observed in the present study.

It is striking that, while exposure to platinum alone results only in a single type of “sluggish” individuals, the combination of platinum and elevated temperature generates phenotypes displaying a whole continuum of shell forms ranging from completely internal shells to “partly-shelled” animals of different morphology, and normally shelled animals. Consequently, the higher temperature must be assigned to be responsible for the re-start of the differential growth of the mantle edge and the shell gland. The higher the temperature the faster is embryonic development in the exotherm species Marisa cornuarietis [7] - this effect could also be observed in our experiments. Actually, the animals exposed to platinum at higher temperatures (28°C - 30°C) exhibited a lower mortality rate than at the lower temperature (26°C).

Heat stress, but also heavy metal exposure and other kinds of proteotoxic stress, induce stress protein synthesis (reviewed in [36]). However, constitutive levels of stress proteins are also important in stabilizing normal embryonic development [37,38] but apparently need to be elevated under proteotoxic condition to enable a proper embryogenesis. Gunter and Degnan [39] exposed embryos of Haliotis asinina to slightly elevated temperatures and observed larvae with abnormally located tissues, although the tissues themselves kept their unique stress protein expression patterns.

There are several examples showing that an induction of stress proteins during embryonic development can reduce the disrupting effect of teratogenic agents. In Drosophila melanogaster an elevated Hsp70 level either caused by a preceding heat shock or by genetic manipulation, mitigates the toxic effects of the mitotic poisons vinblastine and colchicine [40]. Hsp70 and Hsp90, and probably other stress-induced proteins as well, are involved in the cell cycle and in proliferating cells higher Hsp levels can be detected than in cells at another stage of the cell cycle (reviewed in [41]). Hunter and Dix [42] found that in mouse embryos stress-induced Hsp70 was able to decrease the incidence of neural-tube malformations induced by the metalloid arsenite. A further study on human cells by Barnes et al. [43] revealed protection from arsenite-induced genotoxic effects by elevated Hsp70 levels. Osterauer et al. [13] investigated Hsp70-levels in juvenile platinum-exposed Marisa cornuarietis individuals. The highest concentration tested was 100 μg/L PtCl_2_, half of the concentration that was used in this study and, although histological investigations showed histopathological tissue alterations, the Hsp70 level was not significantly elevated. Also own preliminary studies on the induction of stress protein in platinum- and platinum-plus-heat-exposed Marisa embryos did not reveal a consistend induction pattern so far which could have been indicative for a protective role of chaperones in the context of shell gland and mantle edge outgrowth. Thus, for the moment, the role of Hsps in the signalling cascade triggering differential growth in Marisa embryos remains unclear.

In Marisa, the evidently different phenotypes belong in fact to a continuum of gradual morphological variation and result from a more or less intense phenotypic expression of a single developmental trait: the formation and differential growth of an exterior mantle, and, consequently, an exterior shell. The further the exterior mantle is developed, the more “normal” is the development of the snail. This means, that, in this study, elevated temperatures, which are usually considered stressful, actually lead to a more normal development than lower temperatures when interacting with a developmental disruptor, platinum. Together with the observation that mortality in the platinum expositions was lower at higher temperatures than at 26°C this leads to the conclusion that the increase in temperature in this case was not posing additional stress but actually protected development to some extent. There is also another group of stress-induced proteins that should be discussed regarding their possible interaction with platinum: metallothioneins, proteins that are involved in metal trafficking and detoxification. Serafim et al. [44] observed that heavy metal-induced metallothionein levels were higher at higher temperatures in Mytilus galloprovincialis, and Piano et al. [45] even found a temperature-induced increase of metallothionein levels in the absence of heavy metal exposure in the oyster Ostrea edulis. Metallothioneins have also received attention as possible cytosolic sinks for platinum-containing anticancer drugs [46]. Possibly, heat-induced metallothioneins may scavenge platinum in Marisa and thus decrease the internal concentration of “active” platinum. It is, however, unlikely that solely a quantitative reduction of “free” platinum is responsible for the formation of various “partial” shells, because these phenotypes never occurred in exposure experiments with lower platinum concentrations in the medium [10].

Internal shells have evolved in a number of gastropod and other molluscan clades independently from one another. So the question arises, whether there are any similarities between Marisa’s platinum-induced shell internalization and the formation of internal shells which have evolved in some molluscan taxa. In some groups (e.g. Naticidae), developing individuals start with an external shell which is then overgrown by extensions of the foot, the mantle, or both [47]. In Opisthobranchia, shell-internalization and reduction is very common and is theorized to have arisen independently in the different subgroups [48]. In Stylocheilus longicauda, for example, the juvenile shell is overgrown by the parapodia and then shed [49]. The Nudibranchia all lose their juvenile shells and the visceral sac is overgrown by the mantle [50], whereas the Saccoglossa are a heterogenous group with shell-bearing and non-shelled groups [48]. However, in non-shelled Saccoglossa it is not the the mantle that overgrows the visceral sac, it is material of the foot that covers the naked visceral sac [50]. Another gastropod group without external shells is the group of terrestrial pulmonate slugs. In the slugs Arion subfuscens [51] and Limax maximus [52] the shell develops inside a shell sac which is located directly under the dorsal mantle region where it lies horizontally [53]. Slugs, with occasional exceptions like the jumping-slugs of the genus Hemphillia, have a completely internal shell, but they have an external mantle unlike the “sluggish” phenotype of Marisa cornuarietis. Apart from pulmonate slugs, all gastropods have, at least at one time of their lives, external shells which may then be internalized during further development. In “sluggish” Marisa this is similar but less obvious: the first part of their shells, in this study called “plate”, is secreted on the ventral part of the visceral sac and is never covered directly by any tissue but is only hidden from sight by the foot. Only the later formed part of the internal shell is covered by the lobe and calcium carbonate is secreted internally.

Gastropods are not the only molluscan group in which shell reduction and internalization have evolved convergently. Kröger et al. [54] described several different reduction mechanisms in cephalopods, e.g. demineralisation of the shell or reduction of organic compounds. All these examples show that there is a phylogenetic continuum running between the two extrema - shell-bearing and non-shelled - and that different groups have evolved different mechanisms to reach their respective stages in this continuum independently from each other. While the presence or absence of an external shell is a very obvious trait in molluscs, it does not seem to be, mechanistically, a very conserved one. However, there is also a similarity between the platinum-induced shell internalization in Marisa and the internal shell of cephalopods. In some coleoids a second layer of prismatic material is deposited on top of the original one [55]. According to Bandel [56] mineralization of the primary shell occurs both inside and outside the shell and the calcium carbonate is attached to the periostracum from the exterior and the interior. This means that in these animals a part of the shell-producing tissue faces inwards just like it does in “sluggish” and “partly-shelled” Marisa individuals in which the shell gland is located on a lobe. Assuming, that the tissue of this lobe is in fact the tissue that would normally form the mantle, the shell in “sluggish”İ and “partly-shelled” Marisa individuals lies both on the mantle and is, at least partly, covered by it. This condition is similar to the one found in molluscs in which the mantle overgrows the shell, resulting in an inwardly facing shell-secreting tissue, like in almost all extant cephalopods.

## Conclusion

In Marisa, manipulating the growth of the shell gland and the mantle edge during embryogenesis can induce the formation of normal, intermediate and internal shells. These intermediate and internal shells share characteristics with internal shells found in other molluscan groups: the first part of the shell is external and the shell is then partly overgrown. Also, the shell lies on the shell-secreting tissue and is partly covered by it like in many cephalopods. Our experiments with the ramshorn snail Marisa cornuarietis show that the transition from external shell to internal shell does not have to take place gradually via intermediate forms, although these exist, but that a full transition can be caused by a single trigger which specifically arrests differential growth of disctinct tissues.

## Methods

“Sluggish” M. cornuarietis were reared according to the method described by Osterauer et al. [2] and Marschner et al. [3]. “Partly-shelled” M. cornuarietis were reared likewise, but at higher temperatures. All animal care regulations and legal requirements were adhered to.

### Rearing of “sluggish” and “partly-shelled” M. cornuarietis

On the first day of the experiment, freshly deposited egg clutches were removed from aquaria of a Marisa laboratory hatchery and the single eggs were separated with a razor blade. The eggs were then transferred to Petri dishes in a way that all Petri dishes held between 20 and 30 eggs from at least three different clutches per dish. For the platinum exposure experiments, which lead to almost 100% “sluggish” individuals with internal shells, 200 μl platinum chloride standard solution (platinum standard, Ultra Scientific, Wesel, Germany, 1,000 μg/ml, Matrix: 98% water, 2% HCl) were mixed with 1 L tap water from the aquaria resulting in a concentration of 139 μg Pt^2+^/L. For the controls tap water from the aquaria was used. Petri dishes were kept in a climate chamber at 26°C at a light-dark regime of 12:12 h. To obtain “partly-shelled” animals, the Petri dishes with the platinum solution were kept at 28, 29 or 30°C in climate chambers with a light-dark regime of 12:12 h. All test solutions were changed daily. A number of individuals from both the platinum-exposure and the control were transferred to aquarium water after completion of embryonic development, and raised to adulthood in glass Petri dishes and, later, in glass bowls. The water was changed every other day and the animals were fed small portions of Nutrafin Max flakes (Hagen, Germany) and, sometimes, small pieces of carrots. They were kept in a climate chamber at 26°C. “Partly-shelled” animals from the platinum-plus-heat-exposure groups were also transferred to aquarium water after completion of their embryonic development and then kept at 26°C. The development of the snails was photographically documented. All images were edited in Gimp (scaling, rotating, cropping, and color adjusting), labeling was added in Inkscape. Sketches were also done in Inkscape or drawn by hand and modified in Inkscape.

### Scanning electron microscopy

Three fresh egg clutches were selected, severed and distributed into 2 Petri dishes containing platinum solution and one Petri dish with aquarium water. Every Petri dish contained 25-30 eggs which were kept at 30°C. Depending on the number of living embryos and the respective stages of development, embryos were removed from their egg capsules with two syringes and transferred into snap-cap vials filled with fixative (2% glutardialdehyde (VWR-Merck) dissolved in 0.01 M cacodylate buffer (VWR-Merck), pH 7.4). The embryos were selected in a way that for every clutch embryos from subsequent days of development could be obtained. Fixation took place between days 4 and 13 of embryonic development. This period had been identified as the time span in which the development from initially “sluggish” to “partly-shelled” individuals takes place. The embryos were then further processed for SEM imaging by rinsing them in 0.01 M cacodylate buffer (3 × 30 minutes). Subsequently, they were incubated overnight in a solution of reduced osmium tetroxide (2 mL of a solution of 1 g osmium tetroxide and 25 mL aqua dest + 2 mL aqua bidest + 4 mL of potassium ferrocyanide (K_4_[Fe(CN)_6_]*3H_2_O, Merck) and rinsed again in 0.01 M cacodylate buffer (3 × 30 minutes). Samples were then successively dehydrated in 70%, 80%, 90%, 96%, and absolute ethanol (30 minutes for each concentration). Finally, animals were critical point dried, sputtered with gold, and mounted on stubs. Examination took place with a scanning electron microscope (Zeiss Evo LS10).

### Histology

Embryos were removed from their egg capsules and transferred into snap-cap vials filled with fixative. The pictures of “sluggish” embryos in Figure 2 are unpublished data from experiments that have been described by Marschner et al. [3]. Embryos from the platinum exposures at 28°C and 29°C were removed at 12 and 17 days after oviposition. Both, embryos that remained “sluggish” and those with a clearly recognizable “partial” shell, were selected and fixed in 2% glutardialdehyde dissolved in 0.01 M phosphate buffer (pH 7.4). They were then rinsed in phosphate buffer (2 × 30 minutes) and decalcified in 5% trichloroacetic acid in 37% formol (3 × within 24 h). Subsequently, the specimens were dehydrated in a graded series of ethanol (70% for 1 h, 80% for 1 h, 90% for 1 h, 96% for 30 minutes, and 100% for 2 h), and embedded in Technovit (Heraeus Kulzer, Germany). Serial sections of 3 to 3.5 μm thickness were cut with an automatic microtome (2050 Supercut, Reichert-Jung, Germany). Sections were mounted on microscopic slides and stained with hematoxylin/eosin or a modified Mallory’s triple stain (Cason, 1950, modified for Technovit by staining for 1.5 h). A several months old “sluggish” snail and a six-week old control snail were fixed in 2% glutardialdehyde (VWR-Merck) in 0,01 M cacodylate buffer (VWR-Merck) at pH 7.4. Samples were rinsed 3 × in 0.01 M cacodylate buffer (VWR-Merck), decalcified in a mixture of 37% formol and 70% ethanol (1:1) first for 30 minutes and again overnight. They were rinsed in 70% ethanol again and dehydrated in a graded series of 70% (30 minutes and 1.5 h), 80% (1 h), 90% (1 h), 96% (1 h), and 100% ethanol (2 × 1 h). Subsequently, samples were embedded in paraffin and cut in serial sections of 5 μm thickness using a microtome (Leica SM 2000R). The sections were mounted on slides and stained with Mallory’s triple stain [57]. All slides were examined with a light microscope (Axioskop 2, Zeiss, Germany).

## Competing interests

The authors declare that they have no competing interests.

## Authors’ contributions

LM participated in the histological examinations of both adult snails and embryos, the scanning electron microscopy, the data analysis and prepared the manuscript. JS carried out the histological examinations of platinum-and-heat-exposed embryos and SS performed the scanning electron microscopy. RT participated in data analysis and helped draft the manuscript. HRK participated in designing the study and helped with the data analysis and drafting of the manuscript. All authors read and approved the final manuscript.

## References

[B1] West-Eberhard MJ Developmental Plasticity and Evolution 2003 Oxford: University Press

[B2] Osterauer R Marschner L Betz O Gerberding M Sawasdee B Cloetens P Haus N Sures B Triebskorn R Köhler HR Turning snails into slugs: induced body plan changes and formation of an internal shell Evol Dev 2010 12 5 474 483 10.1111/j.1525-142X.2010.00433.x 20883216

[B3] Marschner L Triebskorn R Köhler HR Arresting mantle formation and redirecting embryonic shell gland tissue by platinum2+ leads to body plan modifications in Marisa cornuarietis, (Gastropoda, Ampullariidae) J Morphol 2012 273 8 830 841 10.1002/jmor.20019 22467435

[B4] Crofts DR The development of Haliotis tuberculata with special reference to organogenesis during torsion Philos Trans R Soc London. Ser B, Biol Sci 1937 228 552 219 268 [http://www.jstor.org/stable/92284] [ArticleType: research-article / Full publication date: Oct. 15, 1937 / Copyright Ⓒ1937 The Royal Society] 10.1098/rstb.1937.0012

[B5] Page LR Modern insights on gastropod development: Reevaluation of the evolution of a novel body plan Integr Comp Biol 2006 46 2 134 143 [http://icb.oxfordjournals.org/content/46/2/134.abstract] 10.1093/icb/icj018 21672730

[B6] Aktipis SW Giribet G Lindberg DR Ponder WF Lindberg DR., Ponder WF, Lindberg DR. Gastropoda Phylogeny and Evolution of the Mollusca 2008 Berkeley: University of California Press

[B7] Demian ES Yousif F Embryonic development and organogenesis in the snail Marisa cornuarietis (Mesogastropoda: Ampullariidae). I General outlines of development Malacologia 1973 12 123 150 [PMID: 4736967] 4736967

[B8] Garstang W The origin and evolution of larval forms Nature 1928 122 366

[B9] Page LR Gastropod ontogenetic torsion: developmental remnants of an ancient evolutionary change in body plan J Exp Zoo 2003 297B 11 26 10.1002/jez.b.12 12955840

[B10] Osterauer R Haus N Sures B Köhler HR Uptake of platinum byzebrafish (Danio rerio) and ramshorn snail Marisa cornuarietis and resulting effects on early embryogenesis Chemosphere 1979 77 7 975 982 [http://www.ncbi.nlm.nih.gov/pubmed/19796790] [PMID: 6790] 19796790 10.1016/j.chemosphere.2009.08.033

[B11] Sawasdee B Köhler HR Embryo toxicity of pesticides and heavy metals to the ramshorn snail, Marisa cornuarietis (Prosobranchia) Chemosphere 1927 75 11 1539 1547 [http://www.ncbi.nlm.nih.gov/pubmed/19278713][PMID: 8713] 19278713 10.1016/j.chemosphere.2009.01.085

[B12] Sawasdee B Köhler HR Metal sensitivity of the embryonic development of the ramshorn snail Marisa cornuarietis (Prosobranchia) Ecotoxicology 2010 [http://www.ncbi.nlm.nih.gov/pubmed/20711673][PMID: 20711673] 10.1007/s10646-010-0534-8 20711673

[B13] Osterauer R Köhler HR Triebskorn R Histopathological alterations and induction of Hsp70 in ramshorn snail Marisa cornuarietis and zebrafish embryos after exposure to PtCl2 Danio rerio Aquat Toxicol 2010 99 100 107 [http://www.ncbi.nlm.nih.gov/pubmed/20444508][PMID: 20444508] 10.1016/j.aquatox.2010.04.001 20444508

[B14] Demian ES Yousif F Embryonic development and organogenesis in the snail Marisa cornuarietis (Mesogastropoda: Ampullariidae). II. Development of the alimentary system Malacologia 1973 12 151 174 [PMID: 4718483] 4718483

[B15] Demian ES Yousif F Embryonic development and organogenesis in the snail Marisa cornuarietis (Mesogastropoda: Ampullariidae). 3 Development of the circulatory and renal systems Malacologia 1973 12 2 175 194 [PMID: 4788265] 4788265

[B16] Demian ES Yousif F Embryonic development and organogenesis in the snail Marisa cornuarietis (Mesogastropoda: Ampullariidae). IV. Development of the shell gland, mantle and respiratory organs Malacologia 1973 12 2 195 211 [PMID: 4788266] 4788266

[B17] Demian ES Yousif F Embryonic development and organogenesis in the snail Marisa cornuarietis (Mesogastropoda: Ampullariidae). V Development of the nervous system Malacologia 1975 15 29 42 [PMID: 1221226] 1221226

[B18] Cather JN Cellular interactions in the development of the shell gland of the gastropod, Ilyanassa J Exp Zool 1967 166 2 205 223 10.1002/jez.1401660204/abstract 6080551

[B19] McCain ER Cell interactions influence the pattern of biomineralization in the Ilyanassa obsoleta (Mollusca) embryo Dev Dyn 1992 195 3 188 200 [PMID: 1301083] 10.1002/aja.1001950305 1301083

[B20] Dictus WJ Damen P Cell-lineage and clonal-contribution map of the trochophore larva of Patella vulgata (Mollusca) Mech Dev 1997 62 2 213 226 [PMID: 9152012] 10.1016/S0925-4773(97)00666-7 9152012

[B21] Ponder WF Lindberg DR Towards a phylogeny of gastropod molluscs: an analysis using morphological characters Zool J Linn Soc 1997 119 2 83 265 10.1111/j.1096-3642.1997.tb00137.x

[B22] Haszprunar G On the origin and evolution of major gastropod groups, with special reference to the streptoneura J Mollus Stud 1988 54 4 367 441 10.1093/mollus/54.4.367

[B23] Wolpert L Principles of Development 2011 New York: Oxford: Oxford University Press

[B24] Grande C Patel NH Nodal signalling is involved in left–right asymmetry in snails Nature 2008 457 7232 1007 1011 [http://www.nature.com/doifinder/10.1038/nature07603] 19098895 10.1038/nature07603 PMC2661027

[B25] Jackson DJ WÃűrheide G Degnan BM Dynamic expression of ancient and novel molluscan shell genes during ecological transitions BMC Evol Biol 2007 7 160 [http://www.biomedcentral.com/1471-2148/7/160] 10.1186/1471-2148-7-160 17845714 PMC2034539

[B26] Nederbragt AJ van Loon AE Dictus WJ Expression of Patella vulgata orthologs of engrailed and dpp-BMP2/4 in adjacent domains during molluscan shell development suggests a conserved compartment boundary mechanism Dev Biol 2002 246 2 341 355 [http://linkinghub.elsevier.com/retrieve/pii/S0012160602906536] 10.1006/dbio.2002.0653 12051820

[B27] Arendt D Nübler-Jung K Dorsal or ventral: similarities in fate maps and gastrulation patterns in annelids, arthropods and chordates Mech Dev 1997 61 1-2 7 21 [http://www.sciencedirect.com/science/article/pii/S092547739600620X] 10.1016/S0925-4773(96)00620-X 9076674

[B28] Baratte S Andouche A Bonnaud L Engrailed in cephalopods: a key gene related to the emergence of morphological novelties Dev Genes Evol 2007 217 5 353 362 [http://www.springerlink.com/index/10.1007/s00427-007-0147-2] 10.1007/s00427-007-0147-2 17394016

[B29] Gilbert SF Singer SR Developmental Biology 2006 Sunderland: Palgrave Macmillan

[B30] Hanna LA Peters JM Wiley LM Clegg MS Keen CL Comparative effects of essential and nonessential metals on preimplantation mouse embryo development in vitro Toxicology 1997 116 1-3 123 131 [PMID: 9020513] 10.1016/S0300-483X(96)03534-2 9020513

[B31] Snow ET Metal carcinogenesis: mechanistic implications Pharmacol Ther 1992 53 31 65 [http://www.ncbi.nlm.nih.gov/pubmed/1641401] [PMID: 1641401] 10.1016/0163-7258(92)90043-Y 1641401

[B32] Gebel T Lantzsch H Pleßow K Dunkelberg H Genotoxicity of platinum and palladium compounds in human and bacterial cells Mutat Res/Genet Toxicol Environ Mutagen 1997 389 2–3 183 190 [http://www.sciencedirect.com/science/article/pii/S1383571896001453] 10.1016/s1383-5718(96)00145-3 9093382

[B33] Migliore L Frenzilli G Nesti C Fortaner S Sabbioni E Cytogenetic and oxidative damage induced in human lymphocytes by platinum, rhodium and palladium compounds Mutagenesis 2002 17 5 411 417 [PMID: 12202629] 10.1093/mutage/17.5.411 12202629

[B34] Nordlind K Further studies on the ability of different metal salts to influence the DNA synthesis of human lymphoid cells Int Arch Allergy Immunol 2009 79 1 83 85 10.1159/000233947 3941015

[B35] Osterauer R Faßbender C Braunbeck T Köhler HR Genotoxicity of platinum in embryos of zebrafish Danio rerio and ramshorn snail Marisa cornuarietis Sci Total Environ 2011 409 11 2114 2119 [http://linkinghub.elsevier.com/retrieve/pii/S0048969711001203] 10.1016/j.scitotenv.2011.01.060 21420724

[B36] Gupta SC Sharma A Mishra M Mishra RK Chowdhuri DK Heat shock proteins in toxicology: How close and how far? Life Sci 2010 86 11-12 377 384 [http://www.sciencedirect.com/science/article/pii/S0024320510000044] 10.1016/j.lfs.2009.12.015 20060844

[B37] Rutherford SL Lindquist S Hsp90 as a capacitor for morphological evolution Nature 1998 396 6709 336 342 [PMID: 9845070] 10.1038/24550 9845070

[B38] Queitsch C Sangster TA Lindquist S Hsp90 as a capacitor of phenotypic variation Nature 2002 417 6889 618 624 [PMID: 12050657] 10.1038/nature749 12050657

[B39] Gunter HM Degnan BM Impact of ecologically relevant heat shocks on Hsp developmental function in the vetigastropod, Haliotis asinina J Exp Zool B Mol Dev Evol 2008 310 5 450 464 [http://www.ncbi.nlm.nih.gov/pubmed/18421770] [PMID: 18421770] 18421770 10.1002/jez.b.21217

[B40] Isaenko OA Karr TL Feder ME Hsp70 and thermal pretreatment mitigate developmental damage caused by mitotic poisons in Drosophila Cell Stress Chaperones 2002 7 3 297 308 [http://www.ncbi.nlm.nih.gov/pmc/articles/PMC514829/] [PMID: 12482205 PMCID: PMC514829] 10.1379/1466-1268(2002)007<0297:HATPMD>2.0.CO;2 12482205 PMC514829

[B41] Helmbrecht K Zeise E Rensing L Chaperones in cell cycle regulation and mitogenic signal transduction: a review Cell Prolif 2000 33 6 341 365 [PMID: 11101008] 10.1046/j.1365-2184.2000.00189.x 11101008 PMC6496586

[B42] Hunter ES Dix DJ Heat shock proteins Hsp70-1 and Hsp70-3 are necessary and sufficient to prevent arsenite-induced dysmorphology in mouse embryos Mol Reprod Dev 2001 59 3 285 293 10.1002/mrd.1033/abstract 11424214

[B43] Barnes J Collins B Dix D Allen J Effects of heat shock protein 70 (Hsp70) on arsenite-induced genotoxicity Environ Mol Mutagen 2002 40 4 236 242 10.1002/em.10116 12489113

[B44] Serafim M Company R Bebianno M Langston W Effect of temperature and size on metallothionein synthesis in the gill of Mytilus galloprovincialis exposed to cadmium Mar Environ Res 2002 54 3-5 361 365 [http://www.sciencedirect.com/science/article/pii/S0141113602001216] 10.1016/S0141-1136(02)00121-6 12408589

[B45] Piano A Valbonesi P Fabbri E Expression of cytoprotective proteins, heat shock protein 70and metallothioneins, in tissues of Ostrea edulis exposed to heat andheavy metals Cell Stress Chaperones 2004 9 2 134 142 [http://www.ncbi.nlm.nih.gov/pmc/articles/PMC1065293/] [PMID: 15497500 PMCID: PMC1065293] 10.1379/483.1 15497500 PMC1065293

[B46] Knipp M Metallothioneins and platinum(II) anti-tumor compounds Curr Med Chem 2009 16 5 522 537 10.2174/092986709787458452 19199919

[B47] Ponder WF Colgan DJ Healy JM NÃĳtzel A Simone LRL Strong EE Ponder W F, Ponder W F, Lindberg DR Caenogastropoda Phylogeny and evolution of the Mollusca 2008 Berkeley: University of California Press

[B48] Wägele H Klussmann-Kolb A Opisthobranchia (Mollusca, Gastropoda) – more than just slimy slugs. Shell reduction and its implications on defence and foraging Front Zool 2005 2 3 [http://www.ncbi.nlm.nih.gov/pmc/articles/PMC554092/] [PMID: 15715915 PMCID: PMC554092] 10.1186/1742-9994-2-3 15715915 PMC554092

[B49] Switzer-Dunlap M Hadfield MG Observations on development, larval growth and metamorphosis of four species of Aplysiidae (GastropodaOpisthobranchia) in laboratory culture J Exp Mar Biol Ecol 1977 29 3 245 261 [http://www.sciencedirect.com/science/article/pii/0022098177900697] 10.1016/0022-0981(77)90069-7

[B50] Schmekel L Portmann A Opisthobranchia des Mittelmeeres: Nudibranchia und Saccoglossa 1982 Berlin: Springer-Verlag

[B51] Kükel K Zur Biologie der Lungenschnecken: Ergebnisse vieljÃďhriger Züchtungen und Experimente 1916 Heidelberg: Winter

[B52] Simpson GB Anatomy and physiology of Polygyra albolabris and Limax maximus and Embryology of Limax maximus Albany University of the State of New York Cornell University Library 1901 [http://archive.org/details/cu31924001030612]

[B53] Furbish DR Furbish WJ Structure, crystallography, and morphogenesis of the cryptic shell of the terrestrial slug Limax maximus (Mollusca, Gastropoda) J Morphol 1984 180 3 195 211 10.1002/jmor.1051800304/abstract 30037160

[B54] Kröger B Vinther J Fuchs D Cephalopod origin and evolution: A congruent picture emerging from fossils, development and molecules Bioessays 2011 33 8 602 613 10.1002/bies.201100001 21681989

[B55] Nishiguchi MK Mapes RH Lindberg DR, Ponder W F, Ponder W F, Lindberg DR Cephalopoda Phylogeny and Evolution of the Mollusca 2008 Berkeley: University of California Press

[B56] Bandel K Cephalopod shell structure and general mechanisms of shell formation Short Course Geol Ser 1989 5 97 115 [http://www.agu.org/books/sc/v005/SC005p0097/SC005p0097.shtml]

[B57] Cason JE A rapid one-step Mallory-Heidenhain stain for connective tissue Biotech Histochem 1950 25 4 225 226 10.3109/10520295009110996 14782060

